# Outcomes of Early Administration of Cidofovir in Non-Immunocompromised Patients with Severe Adenovirus Pneumonia

**DOI:** 10.1371/journal.pone.0122642

**Published:** 2015-04-15

**Authors:** Se Jin Kim, Kang Kim, Sung Bum Park, Duck Jin Hong, Byung Woo Jhun

**Affiliations:** 1 Division of Pulmonary and Critical Care Medicine, Department of Medicine, The Armed Forces Capital Hospital, Seong-nam, Gyeonggi province, South Korea; 2 Department of Laboratory Medicine, The Armed Forces Capital Hospital, Seong-nam, Gyeonggi province, South Korea; University of Calgary & ProvLab Alberta, CANADA

## Abstract

The benefits of treatment with antiviral therapy for severe adenovirus (AdV) pneumonia are not well established. We described the clinical characteristics and treatment outcomes of early cidofovir treatment of severe AdV pneumonia in non-immunocompromised patients. We retrospectively reviewed the medical records of all patients diagnosed with severe AdV pneumonia between 2012 and 2014. A total of seven non-immunocompromised patients with severe AdV pneumonia were identified, and all isolates typed (*n* = 6) were human AdV-B55. All patients had progressive respiratory failure with lobar consolidation with or without patchy ground glass opacity. Three patients required vasopressors and mechanical ventilation. All patients had abnormal laboratory findings including: leukopenia, thrombocytopenia, or elevated liver enzymes. After admission, all patients received antiviral therapy with cidofovir, and the median time from admission to cidofovir administration was 48 h and median the time from onset of symptoms to cidofovir administration was 7.1 days. After cidofovir administration, complete symptomatic improvement occurred after a median of 12 days and radiographic resolution occurred after a median of 21 days. Consequently, all patients completely improved without complications. Our data suggest that early administration of cidofovir in the course of treatment for respiratory failure as a result of AdV pneumonia in non-immunocompromised patients could be a treatment strategy worth considering, especially in cases of HAdV-55 infection.

## Introduction

Adenoviruses (AdV) are non-enveloped, double-stranded DNA viruses that can cause diseases of the upper and lower respiratory, gastrointestinal, and ocular systems [1]. Respiratory infection caused by AdV in non-immunocompromised patients is usually mild and self-limited. In contrast, in immunocompromised patients, AdV infection can be disseminated and result in a considerable mortality rate [2–4]. Despite the low incidence of serious illness in non-immunocompromised patients, Levin *et al*. reported fatal AdV pneumonia in a previously healthy adult in 1967 [5], and sporadic cases of severe respiratory disease in non-immunocompromised adults have been reported [6–11] since. These reports include several outbreaks of AdV respiratory infections in previously healthy adults in vulnerable populations, such as residents of mental health care centers and military recruits [12–17].

Previous investigations described the clinical characteristics of severe AdV pneumonia as being distinct from those of other causative agents [8, 18, 19], and revealed that several AdV serotypes were associated with severe disease states [6, 19–21]. However, these efforts have not influenced the overall clinical outcomes in cases of severe AdV pneumonia. Currently, there are no approved antiviral agents for the treatment of severe AdV pneumonia, and limited data on the clinical response to antiviral therapy in immunocompromised patients are available [6, 8].

Our institution, a military hospital with the highest referral rate in South Korea, encounters several cases of AdV pneumonia annually, some of which are severe cases [17, 22], thus we have aimed to produce favorable outcomes by applying antiviral therapy upon confirmation of the diagnosis of severe AdV pneumonia. The present study describes in detail the clinical characteristics and favorable treatment outcomes of non-immunocompromised adults who had experienced severe AdV pneumonia and received early cidofovir administration.

## Materials and Methods

### Study subjects

We retrospectively reviewed the medical records of all consecutive patients diagnosed with AdV pneumonia between December 1, 2012 and June 1, 2014 at the Armed Forces Capital Hospital (874 bed referral military hospital) in Gyeonggi province, South Korea. The diagnosis of AdV pneumonia was considered certain when it was associated with the following: 1) lower respiratory and/or systemic symptoms, 2) lung infiltration on chest radiography or computed tomography (CT) scan, and 3) evidence of AdV infection identified by positive multiplex polymerase chain reaction (PCR) for AdV from lower respiratory tract samples, such as sputum, or bronchoalveolar lavage (BAL) fluid.

Only non-immunocompromised adult patients who fulfilled the criteria for severe community-acquired pneumonia, set out in the Infectious Diseases Society of America/American Thoracic Society Consensus Guidelines [23], and admitted to the intensive care unit with progressive respiratory failure, defined as a partial pressure of arterial oxygen (PaO_2_)/fraction of inspired oxygen (FiO_2_) ratio of < 300 mmHg and/or tachypnea (respiration rate >30 breaths/min) [24], were included in the analysis. Criteria for severe community-acquired pneumonia requiring admission to the intensive care unit are one or more major criteria or three or more minor criteria [23]. Major criteria include: 1) invasive mechanical ventilation and 2) requiring vasopressors due to septic shock. Minor criteria include: 1) respiratory rate ≥ 30/min, 2) PaO_2_/FiO_2_ ratio ≤ 250 mmHg, 3) multilobar pneumonia in chest radiographs, 4) decreased level of consciousness, 5) blood urea nitrogen ≥ 20 mg/dL, 6) white blood cells < 4,000/mm^3^, 7) platelets < 100,000/mm^3^, 8) core temperature < 36°C, and 9) hypotension requiring aggressive fluid therapy.

The Institutional Review Board of the Armed Forces Capital Hospital approved this study and permitted review and publication of patient records. All data on the study patients were analyzed anonymously. The requirement for informed consent by individual patients was waived given the retrospective nature of the study.

### Patient management

Patients who presented with acute respiratory symptoms that deteriorated rapidly despite use of appropriate broad-spectrum antibiotics for 2–3 days and/or who had unusual laboratory findings including leukopenia or extrapulmonary symptoms were suspected of having atypical pneumonia [25], and underwent thorough examinations. Infectious etiologies were investigated using peripheral blood, sputum, and BAL fluid utilizing techniques such as staining and microbiological culture for bacteria and *Mycobacterium tuberculosis*; multiplex real time PCR testing for respiratory bacterial agents including *Streptococcus pneumoniae*, *Haemophilus influenza*, *Mycoplasma pneumoniae*, *Chlamydia pneumoniae*, *Legionella*, and *Bordetella*; and multiplex real time PCR testing for respiratory viruses.

### Multiplex real time PCR assay for respiratory viruses

Nucleic acid extraction was performed on the MagNA Pure LC 2.0 (Roche Diagnostics, Mannheim, Germany) using a MagNA Pure LC Nucleic Acid isolation kit I (Roche Diagnostics) according to the manufacturer’s instructions. More than fifty microliters of clinical samples were used for nucleic acid extraction and eluted in 50 μl of elution buffer.

Multiplex real-time PCR was performed to screen for 15 commonly isolated respiratory viral pathogens, including adenovirus, rhinovirus, influenza virus A/B, respiratory syncytial virus A/B, bocavirus, coronavirus 229E/OC43/NL63/HKU1, parainfluenza virus 1/2/3, and metapneumovirus, using a Real-Q RV Detection kit (BioSewoom, Seoul, Korea) on a Roche Light Cycler 480 II instrument (Roche Diagnostics, Mannheim, Germany) according to the manufacturer’s instructions. The presence of specific viral sequences in the reaction was detected by an increase in the FAM, VIC and Cy5 fluorescence from the relevant dual-labeled probe. Five primer/probe sets were used to detect respiratory viruses. The serotype of human-AdV-positive samples was evaluated by sequence analysis using the partial hexon genomic region.

### Antiviral therapy with cidofovir

Cidofovir (Mylan Institutional) was initiated upon confirmation of AdV infection. Cidofovir, at a dosage of 5mg/kg weekly, was administrated in combination with oral probenecid. Hydration consisted of 1–2 L of normal saline before cidofovir infusion and 1–2 L immediately thereafter to prevent renal toxicity. A total dose of 2-g probenecid was given orally 3 h before infusion, and 1 g at 2 and 8 h after the cidofovir infusion. The number of cidofovir courses administered was determined by the attending physician.

## Results

Eleven patients were newly diagnosed with AdV pneumonia and admitted during the study period; all were non-immunocompromised adults. Of these, four patients did not fulfill the criteria for severe community-acquired pneumonia [23], improved clinically with no antiviral therapy, and were excluded from the analysis. Consequently, seven patients with severe AdV pneumonia were analyzed in this study; their baseline characteristics are shown in Table 1.

**Table 1:** Baseline characteristics of study patients.

Characteristics	Patient						
	1	2	3	4	5	6	7
Age (years)	19	20	21	22	20	19	20
Sex	male	male	male	male	male	male	male
BMI (kg/m^2^)	23.7	23.4	22.4	25.5	21.7	32.4	26.5
Smoking	current	never	never	ex-	current	never	current
Onset time	March	April	December	May	April	May	June
Symptoms or signs							
Fever	+	+	+	+	+	+	+
Cough	+	+	+	+	–	+	+
Purulent sputum	+	+	–	+	+	+	+
Blood tinged sputum	+	+	–	–	–	–	+
Nasal obstruction	–	–	–	–	+	–	–
Sore throat	–	–	–	–	+	–	–
Myalgia	–	–	+	–	–	–	+
Diarrhea	–	–	–	+	+	–	+
Dyspnea (> MMRC scale II)	+	+	+	+	+	+	+
Initial vital signs							
Heart rate (beats/min)	107	105	107	93	110	97	104
Respiratory rate (breaths/min)	35	32	20	24	40	39	35
Systolic blood pressure (mmHg)	105	85	95	110	99	118	135
Body temperature (°C)	39.4	39.8	40.0	39.6	40.0	39.6	40.0
SpO_2_ on room air (%)	86	88	90	89	85	90	NA
PaO_2_ on room air (mmHg)	49.4	53.3	54.0	61.5	56.0	58.5	NA
PaO_2_/FiO_2_ ratio (mmHg)	235.2	253.8	257.1	292.9	266.7	278.6	245.0
Need for oxygen application	+	+	+	+	+	+	+
ICU admission	+	+	+	+	+	+	+
Need for vasopressor	+	+	+	–	–	–	–
Need for mechanical ventilation	+	+	+	–	–	–	–
Initial PSI score	94	115	66	42	75	74	75
Initial SOFA score	9	8	8	5	6	6	6
Days from onset of symptoms to admission	4	6	3	7	7	4	5
Antibiotics before admission	3^rd^ cefa	quinolone	–	quinolone	quinolone	–	3^rd^ cefa
Duration of antibiotics before admission	5	3	NA	3	2	NA	4

BMI, body mass index; MMRC, modified medical research council; SpO2, oxygen saturation; PaO2, partial pressure of arterial oxygen; FiO2,fraction of inspired oxygen; ICU, intensive care unit; PSI, pneumonia severity score; SOFA, sequential organ failure assessment; NA, not applicable; +, positive;-, negative.

### Demographic characteristics

All patients were previously healthy young males without underlying conditions. Three (43%) patients were non-smokers. Body mass index (kg/m^2^) of patients varied from 21.7–32.4. The illnesses of most of the patients (*n* = 6) occurred in early spring, while that of one occurred in the winter. Three (43%) patients were from a military training facility and all patients were from different operational areas.

### Clinical presentation

All patients presented with acute respiratory or systemic symptoms, such as fever (*n* = 7), cough (*n* = 6), purulent sputum (*n* = 6), blood-tinged sputum (*n* = 3), and dyspnea (*n* = 7). One patient had concurrent upper respiratory tract symptoms, and three patients had extra-pulmonary symptoms such as diarrhea.

All patients had respiratory distress, with hypoxemia (PaO_2_/FiO_2_ ratio <300 mmHg). Six (86%) patients had tachycardia (>100 beats/min) or tachypnea (>30 breaths/min). Three (43%) patients needed vasopressors and mechanical ventilation. The median duration from onset of symptoms to admission was 5 days. Five (71%) patients received empirical antibiotics including quinolone (*n* = 3) and third-generation cephalosporins (*n* = 2) before admission. The two remaining patients received quinolone (*n* = 1; identified as *patient 3*) and piperacillin (*n* = 1; identified as *patient 6*) on admission.

### Chest CT findings

**Fig 1:**
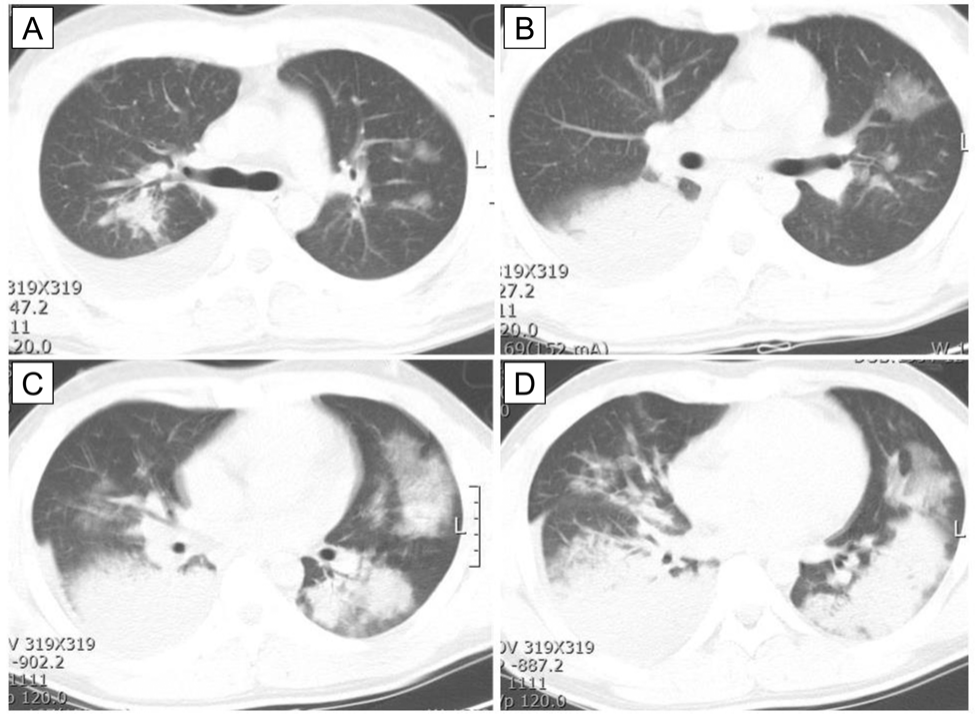
Initial chest CT finding in *patient 1* with severe AdV pneumonia. CT image shows bilateral lobar consolidation (C and D) with ground glass opacity the upper (B) and lower lobes (C and D). Pleural effusion was observed on the right side (A–D).

Initial chest CT findings in patients with severe AdV pneumonia are shown in Table 2. Most patients (*n* = 5, 71%) had bilateral lobar consolidation with ground glass opacity in the upper and lower lobes (Fig 1); only two patients (29%) had unilateral lobar consolidation with or without ground glass opacity. The most commonly involved lobe was the left lower lobe (*n* = 3), followed by the right lower lobe (*n* = 2), left lower lobe (*n* = 1), and both lower lobes (*n* = 1). Pleural effusion was observed in all patients.

**Table 2:** Initial chest CT findings.

Findings	Patient						
	1	2	3	4	5	6	7
Dominant pattern							
Lobar consolidation	+	+	+	+	+	+	+
Patchy ground glass opacity	+	+	–	+	+	+	+
Laterality							
Unilateral	–	–	+	–	–	–	+
Bilateral	+	+	–	+	+	+	–
Lung zone							
Lower	–	–	+	–	–	–	–
Both upper and lower	+	+	–	+	+	+	+
Location of mainly involved lobe							
Left upper lobe	–	–	–	–	–	–	+
Left lower lobe	–	–	+	+	–	+	–
Right lower lobe	–	+	–	–	+	–	–
Both lower lobe	+	–	–	–	–	–	–
Pleural effusion	+	+	+	+	+	+	+

CT, computed tomography; +, positive;-, negative.

### Laboratory findings

The results of initial laboratory tests are shown in Table 3. All patients had thrombocytopenia (<150,000/μL), most (*n* = 6, 86%) patients had leukopenia (<4,000/μL), and none had leukocytosis (>10,000/μL). C-reactive protein levels were elevated in all patients, with a median of 8.31 mg/dL. Procalcitonin levels were elevated in five (71%) patients, with a maximum of 24.23 ng/mL. Hyponatremia was observed in two (29%) patients, and all patients had elevated liver enzymes. Pleural fluid analysis was performed in four (57%) patients; all results were lymphocyte-dominant exudates without evidence of empyema.

**Table 3:** Initial laboratory findings.

Findings	Patient						
	1	2	3	4	5	6	7
Platelet count (/μL)	64,000	122,000	87,000	47,000	95,000	95,000	95,000
Inflammatory markers							
WBC count (/μL)	2,570	2,490	4,240	2,620	1,870	2,550	3,380
Neutrophil (%)	70.1	80.7	77.1	79.7	54.6	66.2	78.4
Lymphocyte (%)	27.6	16.5	17.5	15.3	34.2	27.1	16.3
Neutrophil- lymphocyte ratio	2.5	4.9	4.4	5.2	1.6	2.4	4.8
CRP (mg/dL)	7.22	8.31	13.13	11.67	6.85	5.71	11.87
ESR (mm/h)	3	14	20	15	4	14	14
Procalcitonin (ng/mL)	24.23	0.57	NA	1.41	2.0	0.19	0.79
Hyponatremia	–	+	+	–	–	–	–
Elevated liver enzyme	+	+	+	+	+	+	+
Pleural fluid analysis	Exudate	Exudate	Exudate	NA	Exudate	NA	NA
Neutrophil (%)	3	0	6	NA	2	NA	NA
Lymphocyte (%)	47	39	44	NA	27	NA	NA
Microbiological test							
Gram stain and culture in peripheral blood	–	–	–	–	–	–	–
Gram stain and culture in sputum	–	–	–	–	–	–	–
Gram stain and culture in BAL fluid	–	NA	–	NA	NA	NA	NA
Gram stain and culture in pleural fluid	–	–	–	NA	–	NA	NA
PCR test for bacteria in sputum	–	–	*S*.*pneumoniae*	–	*S*.*pneumoniae H*.*influen*zae	–	–
PCR test for bacteria in BAL fluid	–	NA	NA	NA	NA	NA	NA
PCR test for virus in sputum	AdV	AdV	AdV	AdV	AdV	AdV	AdV
PCR test for virus in BAL fluid	AdV	NA	AdV	NA	NA	NA	NA
Type of AdV	AdV-B55	AdV-B55	NA	AdV-B55	AdV-B55	AdV-B55	AdV-B55

WBC, white blood cell; CRP, C-reactive protein; ESR, erythrocyte sedimentation rate; BAL, bronchoalveolar lavage; PCR, polymerase chain reaction; AdV, adenovirus; NA, not applicable; +, positive;-, negative.

AdV was identified in all patients by PCR in sputum (*n* = 7) and BAL fluid (*n* = 2). Serotyping by sequence analysis was available for six patients; all isolates typed (*n* = 6) were human AdV-B55 (S1 Fig). Bacterial co-infection was identified in the sputum of two (29%) patients by PCR; *S*. *pneumoniae* (*patient 3*) and *S*. *pneumoniae* + *H*. *influenzae* (*patient 5*) were identified. No other causative agent was identified.

### Clinical responses to antiviral therapy in each study patient

**Fig 2:**
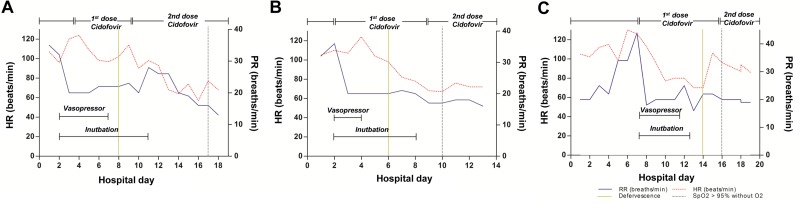
Fig 2 shows the clinical responses of patients 1–3, who needed vasopressor and mechanical ventilation due to septic shock and severe respiratory failure.

**Fig 3:**
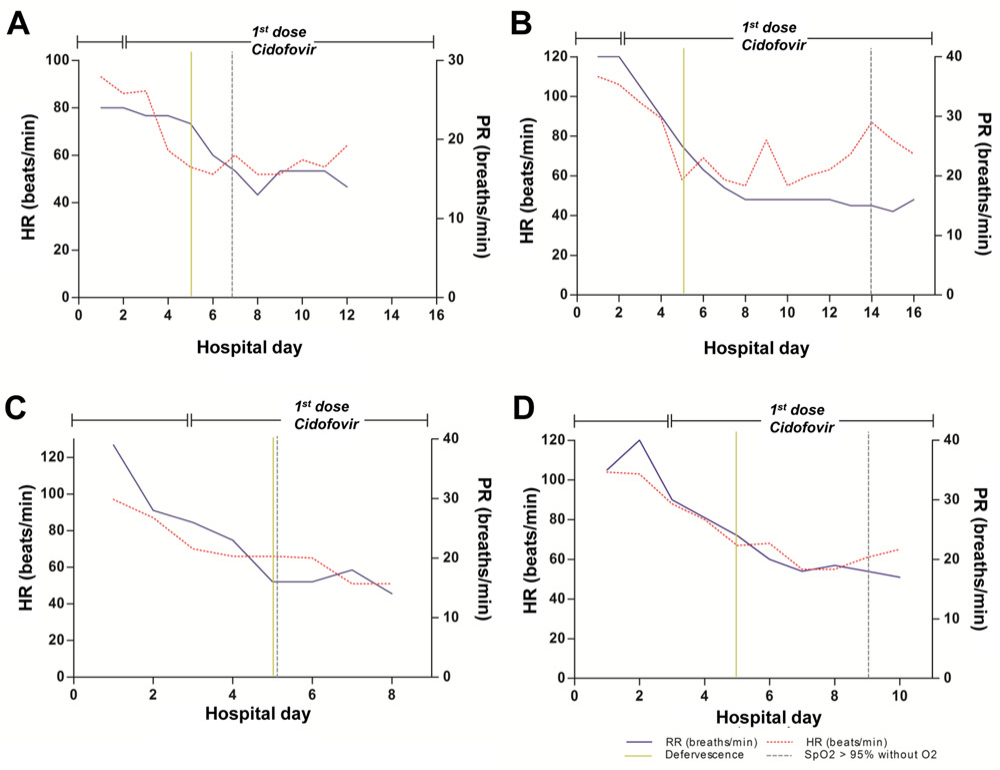
Fig 3 shows the clinical responses of patients 4–7.

The clinical responses to treatment in each patient are shown in Figs 2 and 3. Fig 2 shows the clinical responses of *patients 1–3*, who needed vasopressors and mechanical ventilation due to septic shock and severe respiratory failure. In *patient 1*, after cidofovir administration, blood pressure stabilized after approximately 4 days, and tachypnea, tachycardia, and fever improved within 5 days. Oxygenation completely improved within 14 days (Fig 2A). In *patient 2*, blood pressure stabilized after approximately 2 days, and tachypnea, tachycardia, and fever improved within 4 days. Oxygenation completely improved within 8 days of cidofovir administration (Fig 2B). In *patient 3*, blood pressure stabilized after approximately 4 days, and tachypnea, tachycardia, and fever within 7 days. Oxygenation completely improved within 9 days of cidofovir administration (Fig 2C).

Fig 3 shows the clinical responses of *patients 4–7*. In *patient 4*, tachypnea, tachycardia, and fever improved within 3 days, and oxygenation completely improved within 5 days, of cidofovir administration (Fig 3A). In *patient 5*, tachypnea, tachycardia, and fever improved in 3 days, and oxygenation completely improved within 12 days, of cidofovir administration (Fig 3B). In *patient 6*, tachypnea, tachycardia, and fever improved in 2 days, and oxygenation completely improved within 2 days, of cidofovir administration (Fig 3C). In *patient 7*, tachypnea, tachycardia, and fever improved in 2 days, and oxygenation completely improved within 6 days, of cidofovir administration (Fig 3D).

### Overall treatment outcomes

Overall treatment outcomes of study patients are summarized in Table 4. Median time from admission to identification of AdV infection was 2 (range, 1–6) days. All patients received cidofovir, and most patients (*n* = 6, 86%) received cidofovir within 72 h (minimum, 25 h and median, 48 h); only one patient (14%) received cidofovir 156 h after admission. The median time from onset of symptoms to cidofovir administration was 7.1 days. Three patients (*patients 1*, *2*, *and 3*) who needed vasopressors and mechanical ventilation received two doses of cidofovir. The other four patients (*patients 4*, *5*, *6*, *and 7*) received single doses of cidofovir. Six (86%) patients received adjuvant intravenous immunoglobulin for 3 days.

**Table 4:** Overall treatment outcomes.

Characteristics	Patients						
	1	2	3	4	5	6	7
Time from admission to identification of AdV (days)	2	1	6	1	1	2	2
Time from admission to cidofovir administration (h)	56	26	156	27	25	48	50
Time from onset of symptoms to cidofovir administration (days)	6.3	7.1	9.5	8.1	8	5.1	7.1
Numbers of cidofovir administration	2	2	2	1	1	1	1
Adjuvant IVIG	+	+	+	+	+	–	+
Changes after treatment							
Days from cidofovir to complete symptomatic improvement	14	13	12	10	25	7	10
Days from cidofovir to complete radiographic resolution	40	22	20	19	37	13	21
Days from cidofovir to normalization of WBC count	6	4	NA	7	7	4	3
Days from cidofovir to normalization of CRP	12	13	12	7	6	4	11
Duration of mechanical ventilation (days)	9	6	5	NA	NA	NA	NA
Adverse reaction of cidofovir	–	–	–	–	–	skin rash	–
Total ICU stay (days)	15	10	9	5	6	4	7
Total hospital stay (days)	25	24	40	20	30	15	21
Respiratory sequelae	–	–	–	–	–	–	–
Treatment outcomes	survived	survived	survived	survived	survived	survived	survived
Follow-up PFT within 3 months							
FEV_1_ (%)	98	82	97	87	102	NA	NA
FEV_1_/FVC (%)	92	83	96	106	93	NA	NA

IVIG, intravenous immunoglobulin; WBC, white blood cell; CRP, C-reactive protein; ICU, intensive care unit; FEV_1_, forced expiratory volume in 1 second; FVC, forced vital capacity; NA, not applicable; +, positive;-, negative.

**Fig 4:**
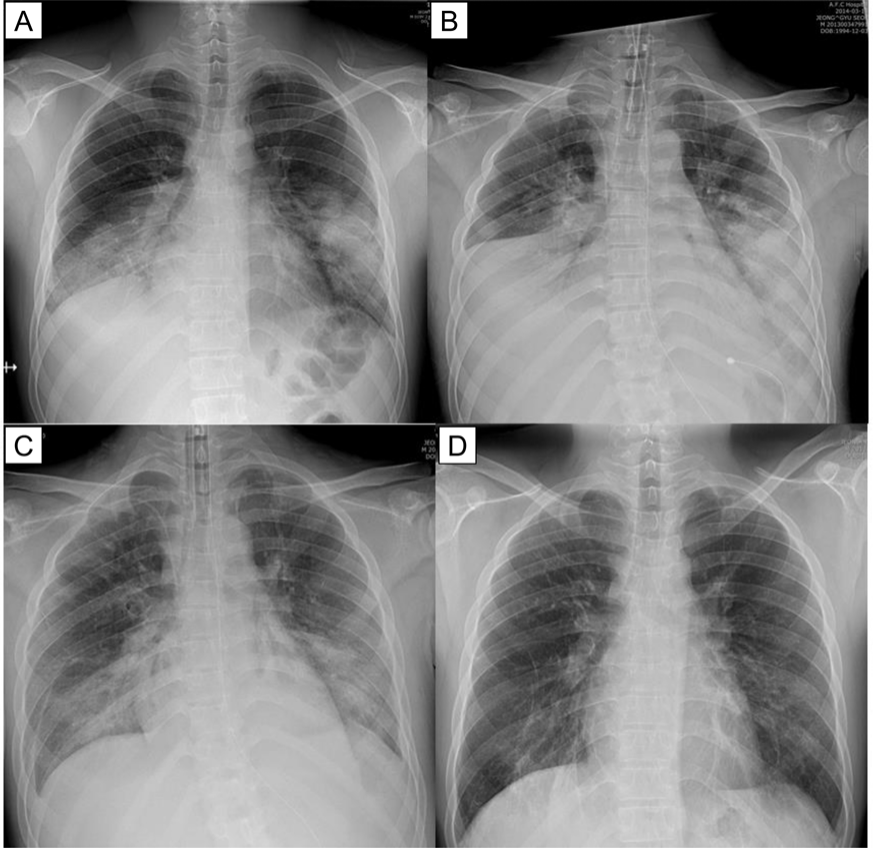
Radiological findings in *patient 1* with severe AdV pneumonia. Chest radiography on admission shows (A) bilateral consolidation in both lower lobes. Next day, the patient was rapidly deteriorated and needed vasopressor and mechanical ventilation (B). Approximately 4 days after cidofovir administration, radiographic resolution occurred (C). Chest radiography obtained after 3 weeks of cidofovir administration shows (D) nearly complete resolution of the previously abnormal radiographic findings.

After cidofovir administration, clinical, radiological, and laboratory improvement occurred in all patients. Complete symptomatic improvement occurred after a median of 12 (range, 7–25) days, and complete radiographic resolution occurred after a median of 21 (range, 13–40) days (Fig 4). Normalization of white blood cell count and C-reactive protein level after a median of 6 (range, 3–11) days and 11 (range, 4–13) days, respectively. Adverse reactions (skin rash) to cidofovir occurred in one patient, and resolved completely with only conservative management. All patients recovered completely without complications.

## Discussion

In the present study, we described favorable outcomes to antiviral therapy with cidofovir in non-immunocompromised adult patients with severe AdV pneumonia. Our data suggest that early administration of cidofovir in the course of respiratory failure could be a treatment strategy worth considering in severe AdV pneumonia. In the seven cases discussed, cidofovir was administered more promptly than in previous fatal cases. In previous studies, most patients did not receive antiviral therapy with cidofovir; indeed, even in the few patients who did receive cidofovir, the administration time was not early in the course of respiratory failure [6, 8, 12] and they had poor outcomes. For example, in a recent study that evaluated outcomes in five patients with severe AdV pneumonia caused by AdV-55 infection in non-immunocompromised adults [26], similar to our study group, four (80%) of the five patients died despite receiving appropriate respiratory support and antiviral therapy, including acyclovir, ganciclovir, and ribavirin, without using cidofovir. In a study of the etiology and epidemiology of acute viral lower respiratory tract infections of South Korean soldiers in our institution, six cases of severe AdV pneumonia were identified. All six patients required mechanical ventilation, most (5/6) did not receive cidofovir, and half (3/6), including one patient who was treated with cidofovir 7 days after admission, died [17]. Moreover, in a case report of fatal AdV pneumonia in immunocompetent adult identical twins, one patient did not recover despite administration of cidofovir antiviral therapy no sooner than 8 days after the onset of symptoms [6]. In the present study, in contrast, time from admission to identification of AdV was short (median, 2 days), and all patients received antiviral therapy on the day of diagnosis of AdV infection. Given this background, our findings may indicate that administration of cidofovir early in the course of respiratory failure may be a beneficial treatment strategy in severe cases of AdV respiratory infection, and this may assist physicians in making decisions regarding diagnosis and treatment.

In our study, all isolates typed (*n* = 6) were human AdV-B55 [27, 28]. To date, more than 60 human AdV types have been identified [29–32], and serotypes 1–5, 7, 11, 14, 21, and 41 are most commonly associated with human disease. Among these, human AdV-B55 has been identified as an emergent acute respiratory disease pathogen, fully characterized by whole-genome sequencing, and has caused two recent outbreaks; one in China in 2006 [33] and one in Singapore in 2005 [14]. In one study that evaluated 48 cases of AdV pneumonia [19], patients infected with human AdV-55 had higher pneumonia severity index scores compared with those infected with other serotypes. Thus, human AdV-55 could be a major pneumonia pathogen in the general population, and further surveillance and monitoring of this agent as a cause of pneumonia are warranted. However, to date, limited data on the correlation between human AdV-55 and clinical severity or clinical courses are available; and no data are available on the treatment responses to cidofovir in cases of human AdV-55 infection. In these contexts, our data may have clinical significance and further studies on the associations of AdV serotypes with severe respiratory infection are necessary.

The pathophysiology of AdV pneumonia remains unclear; few studies have been performed. For example, Prince *et al*. [34] and Gregory *et al*. [35] indicated that much of the acute damage in adenovirus infection appears to be due to dysregulation of the immune system. These features may, in part, explain the self-limiting nature of cases of even severe AdV infection without antiviral therapy, and suggest that the host’s immunological reaction to the infection is a major factor in the pathogenesis of the lung injury. Moreover, this implies that antiviral therapy administered late in the course of respiratory failure may not be effective. However, because there are no accurate data on the pathophysiology of AdV, further studies are needed.

This study had several limitations. Firstly, the study was not controlled with respect to the treatment administered because of its retrospective design. Although we obtained favorable outcomes, other factors may have influenced the outcomes in the study patients, such as the supportive treatment strategies, including ventilator settings and the prone position, or host factors, including young age and previous good health status, and moreover, self-limiting cases of severe AdV infection have been reported previously [9–11, 16]. To date, no controlled study has been reported on the benefits of antiviral therapy in severe AdV pneumonia. This may be mainly because of the rarity of severe AdV pneumonia in non-immunocompromised adults and ethical problems in using antiviral agents that have not obviously been demonstrated to have clinical effect. In this context, our data, despite the limitations, may suggest possible benefits of administering cidofovir as early as possible in severe AdV pneumonia caused mainly by human AdV-55. Based on our clinical data, further controlled studies are warranted to confirm the benefits of cidofovir. Second, despite extensive microbiological testing, the possible influences of bacterial or other co-infection on treatment outcomes cannot be excluded. Furthermore, this study included only a small number of patients. Additionally, the study population was not representative of the general population in terms of age or gender because our study was conducted at a military hospital; thus, large prospective studies are needed.

## Conclusions

We described favorable clinical outcomes to antiviral therapy with cidofovir in non-immunocompromised adult patients with severe AdV pneumonia. Our data suggest that early administration of cidofovir in the course of treatment for respiratory failure as a result of AdV pneumonia in non-immunocompromised patients could be a treatment strategy worth considering, especially in cases of HAdV-55 infection.

## Supporting Information

S1 Fig Examples of AdV sequencing. (TIF)
