# Supplementary figures and images for: Outcomes of Early Administration of Cidofovir in Non-Immunocompromised Patients with Severe Adenovirus Pneumonia
Source: PLoS One. 2015 Apr 15;10(4):e0122642. doi: 10.1371/journal.pone.0122642 (PMC4398328; doi:10.1371/journal.pone.0122642)

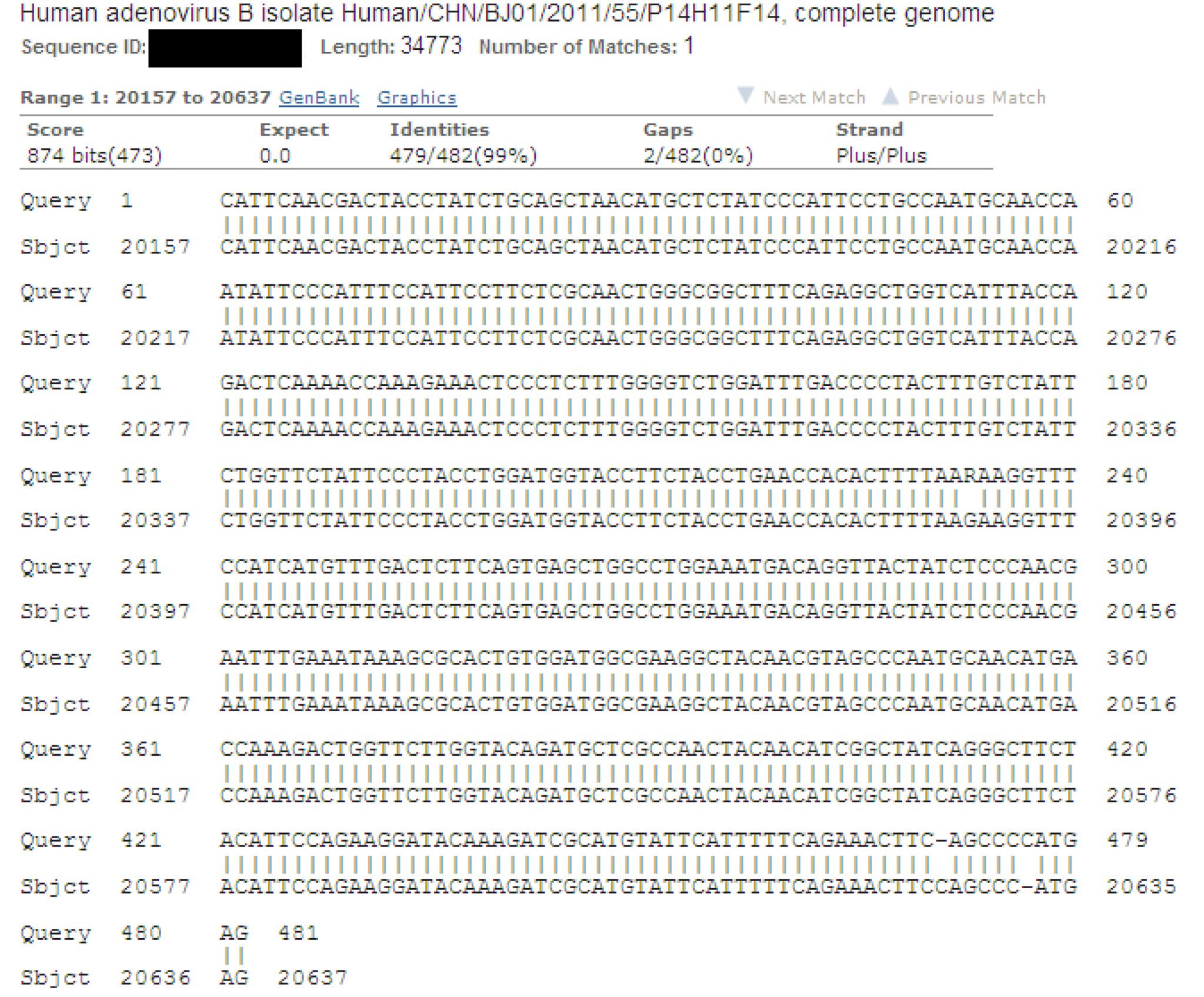

Supplement: S1 Fig — (TIF) [file pone.0122642.s001.tif]
